# Vaccines in civilian defense against bioterrorism.

**DOI:** 10.3201/eid0504.990413

**Published:** 1999

**Authors:** P K Russell

**Affiliations:** Johns Hopkins School of Public Health, Baltimore, MD, USA. biodefen@jhsph.edu


### Vaccines in Civilian Defense Against Bioterrorism

**Philip K. Russell**
Johns Hopkins School of Public Health, Baltimore, Maryland, USA

In the United States, over the past half century, we have lived under the protective umbrella of vaccination programs that shield our population from a dozen serious and sometimes fatal naturally transmitted illnesses. Vaccination has been the single most cost-effective public health intervention. However, the value of vaccines in protecting the population against the deliberate release of infectious organisms is not so clear-cut.

The U.S. armed forces have recognized the military value of vaccines against biological threats and have a long-standing research and development program for a series of vaccines to protect service members from hostile use of a biological agent. Vaccination against anthrax is under way in all three armed services. The Department of Defense has a large program to develop and license additional vaccines for biological defense. For the military, vaccination is an effective means of countering a known threat because the population at risk is easily defined and a high level of vaccine coverage can be achieved.

In evaluating the role of vaccines for protecting the civilian population, quite different answers are reached. Despite the protective efficacy of vaccines against individual organisms, the very high costs and the great difficulties involved in vaccinating large populations, along with the broad spectrum of potential agents, make it impossible to use vaccines to protect the general population against bioterrorism. Thus, vaccines cannot be considered a first line of defense against bioterrorism for the general population, as they can be for the relatively small military population. However, if suitable vaccines can be made available, they have several potential uses: control of a smallpox epidemic and prevention of a global pandemic, postexposure prophylaxis against anthrax (with antibiotics), and preexposure prophylaxis in first-responders at high risk, laboratory workers, and health-care providers.

Address for correspondence: Philip K. Russell, Johns Hopkins Center for Civilian Biodefense Studies, Candler Building, Suite 850, 111 Market Place, Baltimore, MD 21202, USA; fax: 410-223-1665; e-mail: biodefen@jhsph.edu.

Smallpox and anthrax, which pose the greatest risk for causing large numbers of casualties in the event of an effective release by a terrorist group, are at the top of the list of threat agents. Licensed vaccines against both anthrax and smallpox that protect against aerosol transmission are available. An existing licensed plague vaccine is protective against flea-transmitted disease but not against aerosol challenge in animal experiments or against pneumonic plague. This vaccine is in limited supply, and the manufacturer has recently ceased production.

The Department of Defense Joint Vaccine Acquisition Program has several experimental vaccines in development (Table). These vaccines will be further developed and tested with the intent of obtaining products licensed by the U.S. Food and Drug Administration.

Table. Vaccines against biological agents

| Licensed vaccines | Vaccines in research and development |
|---|---|
| Anthrax | Vaccinia (cell culture) |
| Smallpox (vaccinia) | Botulinum toxoids |
| Plague | Tularemia |
| | Q fever |
| | VEE, EEE, WEE |

VEE, Venezuelan equine encephalitis; EEE, Eastern equine encephalitis; WEE, Western equine encephalitis.

### Smallpox

One vaccine in development that is of great importance to civilian biodefense is the vaccinia virus vaccine made in cell culture. A new national stockpile of vaccinia vaccine is urgently needed to respond to the possible threat of a deliberate release of smallpox virus. Even though such release is unlikely, the consequences of being unprepared would be a global catastrophe. An unchecked epidemic in today's unvaccinated, densely packed urban populations linked by rapid air travel could kill millions. The only possible course of action would be to mount a global effort to control the spread and eradicate the disease using vaccinia virus vaccine. The number of deaths due to secondary and subsequent spread of this highly contagious virus would be determined by the rapidity of the public health response, the effectiveness of a vaccination campaign, and, most importantly, the availability of vaccine.

The national stockpile (fewer than 7 million doses of vaccinia virus vaccine) is insufficient to meet national and international needs in this scenario. The stockpile is also deteriorating and has a finite life span. The vaccine was made using the traditional method of scarifying and infecting the flanks and bellies of calves and harvesting the infected lymph. No manufacturer exists today with the capability to manufacture calf lymph vaccine by the traditional method. Replacing the stockpile will require the development and licensure of a new vaccine using modern cell-culture methods. This development program, which will include process development, validation of a new manufacturing process, and extensive clinical testing, will be expensive and may take several years (1).

Obstacles to the development of the vaccine include the lack of satisfactory stocks of vaccinia immune globulin necessary for managing complications of vaccination. Clinical testing cannot proceed without a supply of vaccinia immune globulin. As part of the development effort, the problems associated with manufacture of sufficient quantities of vaccinia immune globulin will have to be addressed and solved. The Department of Defense program is moving ahead with development of a cell-culture vaccine by using a cloned strain of vaccinia derived from another strain. Both civilian and military requirements could be met by a combined and expanded development effort using either the cloned strain or one of the licensed vaccinia strains. The development costs will undoubtedly be high, as for any new biologic product, but the cost of preparedness is insignificant when weighed against the costs of an unchecked smallpox epidemic.

### Anthrax

Anthrax is the second threat that requires a major research and development effort to meet civilian needs. A covert attack, which exposes an urban population to an anthrax spore aerosol, is thought by some to be the most likely scenario for a bioterrorism attack. If the release is detected or the first cases are rapidly diagnosed, rapid action can save many lives. Providing the exposed population with antibiotics followed by vaccination could be lifesaving for exposed persons who would otherwise become ill with untreatable inhalation anthrax in the subsequent few weeks. Prophylactic antibiotics alone will prevent disease in persons exposed to antibiotic-susceptible organisms, but incorporating vaccination into the treatment regime can greatly reduce the length of treatment with antibiotics. Without vaccination, antibiotics must be continued for 60 days; if effective vaccination can be provided, this can be reduced to 30 days. Vaccination of persons affected by an attack will also face the issue of environmental contamination of urban areas after an attack. Stockpiling a vaccine capable of inducing protective immunity with two doses could be extremely valuable in reducing the impact of a terrorist release of anthrax.

The current anthrax vaccine manufactured by Bioport (formerly the Michigan Department of Public Health Laboratory) is an alum-adsorbed, partially purified culture filtrate of *Bacillus anthracis* with a high protective antigen content. The schedule for administration is 0, 2, and 4 weeks and 6, 12, and 18 months. This vaccine is safe and efficacious and is being used by the armed forces to protect personnel against the use of anthrax as a weapon. Immunization of rhesus monkeys followed by a high-dose aerosol challenge has convincingly demonstrated the capability of this vaccine to protect against aerosol challenge with *B. anthracis* spores. The multiple dose requirement, however, is a drawback for civilian use.

Studies in progress may find ways to allow modification of the schedule. Vaccine supply is limited, as is production capacity. As a result, at least for the immediate future, the armed forces will require the entire available supply. This vaccine is made by a method developed before the advent of molecular biology and requires dedicated facilities because *B. anthracis* is a spore-forming organism. In addition to having a multiple-dose requirement, the vaccine is not highly purified and contains multiple extraneous proteins. The characteristics of the vaccine and the constraints on the present method of manufacturing argue strongly against procuring large amounts for civilian use when the technology and the science base exist to rapidly develop a second-generation, improved anthrax vaccine.

Anthrax depends on two toxins (lethal factor and edema factor) for virulence. A protein called protective factor is an essential component of both toxins. The protective factor content is the basis for the effectiveness of the current vaccine. A vaccine based on purified protective factor made by recombinant technology has been protective in animals (2). Use of a modern adjuvant with purified recombinant protective factor should make it possible to have a very effective two-dose vaccine. A recent report of the Institute of Medicine Committee on Research and Development to Improve Civilian Medical Response to Chemical and Biological Terrorism makes a strong case for a major research and development effort leading to an improved second-generation vaccine (1).

Questions regarding the ability of existing anthrax vaccines to protect against anthrax strains engineered to contain additional virulence genes have been raised in Russia (3). Research is needed to address this and related questions about the pathogenesis of anthrax and protective immunity.

The value of vaccinating law-enforcement and emergency response personnel, who must respond to threats (real or otherwise), depends on the nature of their work and the immediacy of the threat. Laboratory personnel who must work with unknown materials and with high concentrations of known infectious materials must be vaccinated. These are additional justifications for moving ahead with a vigorous development program for anthrax and smallpox vaccines.

Dr. Russell is professor, Center for Immunization Research, Johns Hopkins School of Public Health; former Commander, United States Army Medical Research and Development Command.

### References

1. Committee on R&D Needs for Improving Civilian Medical Response to Chemical and Biological Terrorism Incidents. Institute of Medicine, National Academy of Sciences. Chemical and Biological Terrorism. Research and Development to Improve Civilian Medical Response. Washington: National Academy Press; 1999.
2. Zajtchtchuk R, Bellamy RF, editors. Textbook of military medicine: medical aspects of chemical and biological warfare. Office of the Surgeon General, Department of the Army. Washington, D.C.; 1997.
3. Pomeratnsev AP, Startsin NA, Mockov YV, Marnin LI. Expression of cereolysin AB genes in *Bacillus Anthracis* vaccine strain ensures protection against experimental hemolytic anthrax infection. Vaccine 1997;15:1846-50.
